# Explanatory Factors for Disease-Specific Health-Related Quality of Life in Women with Anorexia Nervosa

**DOI:** 10.3390/jcm10081592

**Published:** 2021-04-09

**Authors:** Laura Al-Dakhiel Winkler, Claire Gudex, Mia Beck Lichtenstein, Michael Ejnar Røder, Carol E. Adair, Jan Magnus Sjögren, René Klinkby Støving

**Affiliations:** 1 Center for Eating Disorders, Research Unit for Medical Endocrinology, Odense University Hospital, Mental Health Services in the Region of Southern Denmark, DK-5000 Odense, Denmark; laurawinkler@dadlnet.dk (L.A.-D.W.); rene.stoeving@rsyd.dk (R.K.S.); 2 Department of Clinical Research, University of Southern Denmark, DK-5000 Odense, Denmark; 3 OPEN—Open Patient Data Explorative Network, Odense University Hospital, DK-5000 Odense, Denmark; 4 Centre for Telepsychiatry, Region of Southern Denmark, Department of Clinical Research, University of Southern Denmark, DK-5000 Odense, Denmark; mlichtenstein@health.sdu.dk; 5 Steno Diabetes Center Odense, Odense University Hospital, DK-5000 Odense, Denmark; michael.roder@rsyd.dk; 6 Departments of Community Health Sciences and Psychiatry, Cumming School of Medicine, University of Calgary, Calgary, AB T2N 4N1, Canada; ceadair@ucalgary.ca; 7 Center for Eating Disorders, Ballerup Psychiatric Center, DK-2750 Ballerup, Denmark; jan.magnus.sjoegren@regionh.dk; 8 Institute for Clinical Medicine, University of Copenhagen, DK-2200 Copenhagen, Denmark

**Keywords:** quality of life, anorexia nervosa, female, controls, surveys and questionnaires

## Abstract

A better understanding of explanatory factors for disease-specific health-related quality of life (HRQoL) in anorexia nervosa (AN) could help direct treatment providers to aspects of the most relevance for patient wellbeing and recovery. We aimed to investigate whether factors associated with HRQoL are the same for women with AN and normal-weight controls. The participants in this study were women with AN recruited from specialized eating disorder centers in Denmark and healthy, normal-weight controls invited via online social media. Participants completed online questionnaires on medical history, disease-specific HRQoL (Eating Disorders Quality of Life Scale, EDQLS) and generic HRQoL (SF-36), eating disorder symptomatology, depression, psychological wellbeing, and work and social adjustment. Questionnaires were fully completed by 211 women with AN (median age 21.7 years) and 199 controls (median age 23.9 years). Women with AN had poorer scores on all measures, i.e., worse HRQoL, psychological health, and work/social functioning. Eating disorder symptomatology affected EDQLS score in both groups, but poorer HRQoL in women with AN was also significantly associated with worse scores on bulimia, maturity fears, depression, vitality, and with older age. The factors investigated together explained 79% of the variance in EDQLS score. Management of disordered self-assessment and thought processes may be of particular importance to women with AN. Greater emphasis on these aspects alongside weight gain could enhance patient–clinician alliance and contribute to better treatment outcomes.

## 1. Introduction

The physical and psychological consequences of anorexia nervosa (AN) are severe and affect many aspects of the patient’s life. Treatment requires a multifaceted approach including psychiatric, nutritional, and psychological elements [1], and this has encouraged a change in focus from assessment of purely clinical parameters to inclusion of patient-reported assessments of quality of life [2]. The term health-related quality of life (HRQoL) is used to refer to quality of life aspects related to ill-health, disability, and treatment and is a multidimensional concept that describes physical, social, and psychological wellbeing and functioning [3].

Patient-reported HRQoL is particularly relevant in AN due to the ego-syntonic nature of the disease, and its omission may lead to large discrepancies between what the patient experiences and what the clinician perceives [4]. In another ego-syntonic condition, obsessive–compulsive disorder, the use of patient-reported outcome measures was possibly better than clinical measures in predicting prognosis after treatment [5].

HRQoL measures used in eating disorders were initially generic measures (containing items that would be relevant for any patient group and the general population), but more recently, HRQoL measures have been developed specifically for eating disorders. Both approaches have found HRQoL to be significantly impaired in AN compared to normal reference groups and patients with somatic or other psychiatric illness [2,6]. While generic HRQoL measures are useful for comparative studies, they may be less sensitive to particular aspects of eating disorders [7], and disease-specific measures may be better indicators of ED severity [8].

Several disease-specific questionnaires have been developed for use in AN, such as the Eating Disorders Quality of Life Scale (EDQLS) [9], the Eating Disorders Quality of Life questionnaire (EDQOL) [10], and the Quality of Life for Eating Disorders (QOL ED) [11]. These measures have been developed in different countries and with different goals and characteristics, and so far, none of them have become a gold standard for use in AN. However, the EDQLS was developed and validated in both clinical [9] and non-clinical settings [12] and is available in Danish. Two unique aims for developing the EDQLS were to minimize ego-syntonic responses and to ensure that items were appropriate for completion by adolescents.

The relationship between clinical variables and disease-specific HRQoL appears to be complex. A one-year prospective study using a disease-specific measure (EDQoL) found both baseline BMI and improvement in BMI were associated with better HRQoL in 63 women with severe and enduring AN [13]. In contrast, comorbidity was a more powerful predictor of HRQoL (using the HeRQoLED-short form) than age or BMI in patients with various eating disorders followed over 15 years [14].

Better understanding of the explanatory factors for patient-reported HRQoL in AN would help direct the attention of treatment providers to aspects of most relevance for patients’ wellbeing and recovery. Further, it is currently unknown whether factors associated with HRQoL are the same for women with AN and healthy, normal-weight female controls, or whether some aspects can be identified as having particular importance to women with AN.

The aim of the current study was to explore factors associated with disease-specific HRQoL in AN. We believe this is the largest study of disease-specific HRQoL in AN to date, and the inclusion of a control group enables direct comparisons between women with AN and healthy, normal-weight women. First, we investigated differences between women with AN and healthy female controls in terms of eating disorder pathology, depressive symptomatology, psychological wellbeing, work functioning, and HRQoL assessed using a disease-specific measure (EDQLS) and a generic measure (SF-36). Secondly, we investigated which factors were associated with poorer disease-specific HRQoL in terms of the overall EDQLS score and its subscale scores.

## 2. Materials and Methods

### 2.1. Study Participants

The study participants comprised women with AN and female controls who were recruited as part of a larger cross-sectional study [15].

Female inpatients and outpatients who were diagnosed with AN were recruited from the specialized eating disorder centers in the five regions of Denmark. Diagnoses were established by the clinical staff according to the WHO International Classification of Diseases and Related Health Problems 10th Revision (ICD-10). Comorbid psychiatric disorder was not an exclusion criterion. Information about comorbid psychiatric diagnoses was not collected, but daily use of medication was reported.

The AN patients were invited to participate using an online survey between 14 June 2017 and 10 March 2019. They were compensated EUR 20 for their participation. Inclusion criteria were females with BMI < 18.5 kg/m^2^ and age between 13 and 40 years. Exclusion criteria were age <13 years or ≥40 years, and BMI within the normal range. If participants were below 19 years of age, their BMI was converted into BMI-for-age percentiles according to the WHO’s growth reference [16]. Among adolescents, a BMI-for-age percentile below 10 indicates underweight, while a normal weight range is represented by a BMI-for-age percentile between 10 and 85 [17].

Healthy, normal-weight controls were recruited through online invitations on the social media platform Facebook. An invitation to participate in the study was uploaded on the first author’s media platform and encouraged to be shared among her followers. Inclusion criteria for controls were females aged between 13 and 40 years, and a normal BMI (18.5–24.9). Controls were excluded if they reported psychiatric disorders or used regular medication other than oral contraceptives or vitamins.

When participants accessed the QR code or internet link provided in the invitation, they were automatically directed to a platform for either women with AN or controls as relevant. The first questions determined the participant’s eligibility (inclusion and exclusion criteria) before redirecting them to the first of seven questionnaires. The total time for completion was approximately two hours, and it was possible to save answers and return for completion later if needed. In questionnaires where the phrasing “eating disorder” was used, controls were asked to rephrase this to “eating”.

The data were automatically uploaded to a secure online web application via Open Patient data Explorative Network (OPEN) at Odense University Hospital.

### 2.2. Ethics

The project was approved by the Danish Data Protection Agency (File no. 17/3218) and the Regional Committees on Health Research Ethics for Southern Denmark. The larger cross-sectional study is registered with ClinicalTrials.gov (accessed on 23 February 2021) with the registration number NCT03230435. Participants were thoroughly informed regarding the aim of the study, with legal guardians giving consent for participants under 18 years old.

### 2.3. Questionnaires

Women with AN were asked to complete questions on duration of AN, height, and both nadir and maximum weight. These data were used to calculate self-reported BMI as weight in kg/(height in m)^2^. All participants were asked to provide information about medication, alcohol consumption, drug use, and level of education.

The EDQLS was used as a disease-specific HRQoL questionnaire for eating disorders. It was developed in Canada in 2005 [9] to minimize the effect of ego-syntonic answers of respondents with AN, bulimia nervosa, or eating disorder not otherwise specified. The EDQLS includes 40 items in 12 subscales. Each item is rated on a five-point Likert scale, where higher scores indicate better HRQoL. The total maximum score is 200. The EDQLS has been translated to Danish, with the back translation being checked for congruence to the original connotations in English by the developers [18]. A validation study found excellent internal consistency of the total score of the EDQLS (Cronbach alpha: 0.94), and the scale was received positively in an initial pretest with six patients [15].

The Short-Form 36 (SF-36) comprises 36 questions on physical and emotional health that are summarized into eight subscales, with higher scores indicating better health [19]. The eight subscales are physical functioning, role physical, bodily pain, general health, vitality, social functioning, role emotional, and mental health. The SF-36 has been translated into Danish and validated in a Danish sample [20].

The Eating Disorder Inventory-3 (EDI-3) has 91 items measuring the severity of eating disorder behavior [21]. It has been validated in a Danish sample [21,22]. The 91 items are divided into 12 subscales of drive for thinness, bulimia, body dissatisfaction, low self-esteem, personal alienation, interpersonal insecurities, interpersonal alienation, interoceptive deficits, emotional dysregulation, perfectionism, ascetism, and maturity fears. Items are rated on a four-point scoring system where higher scores indicate a higher level of ED psychopathology.

The Beck Depression Inventory (BDI) [23] has been used in both clinical and research settings since its development in 1961. The total score is based on 21 questions (each rated between 0 and 3), with higher scores indicating more symptoms of depression. A total score of 0–13 indicates minimal depression, 14–19 indicates mild depression, 20–28 indicates moderate depression, and 29–63 indicates severe depression. It has been validated in a Danish sample [24].

Psychological wellbeing was assessed using the WHO-5 wellbeing index (WHO-5) [25,26]. This comprises five items, each rated on a six-point Likert scale, where higher scores indicate greater wellbeing. Total scores are stratified into three groups where a score <35 indicates a risk of psychopathology, a score of 35–50 indicates a lower score than the general population, and a score above 50 is similar to the general population. The WHO-5 was developed in Denmark and has shown adequate validity in a range of clinical areas [27].

The Work and Social Adjustment Scale (WSAS) is a short questionnaire on functional impairment [28]. It comprises five items inquiring about the extent to which a disorder influences the individual’s daily activities. Each item is scored on a nine-point Likert scale. Scores under 10 indicate non-pathological impairment, scores of 10–20 indicate significant impairment, and scores over 20 indicate severe impairment. The WSAS has not been validated in a Danish sample but has been translated into Danish and validated in a Norwegian sample [29].

### 2.4. Statistical Analysis

Descriptive data are presented as medians with interquartile range or as absolute frequencies and percentages. EDQLS domain scores and total scores were summarized and compared by participant group using *t*-tests. Median EDQLS total scores and explanatory variables were compared by the participant group (women with AN vs. controls) using *t*-tests [30]. The association between variables and EDQLS outcome score was investigated using multiple regression analyses with bootstrapped standard errors (500 replications) in two models: Model I for women with AN and Model II for controls. For the regression analyses, R^2^ was reported to determine the amount of explained variance in the EDQLS ratings. We subsequently performed regression analysis including the EDI-3 subscales to investigate the explanatory power of each subscale on EDQLS. As depression could be an important confounding factor in these associations, we controlled for this by including the total BDI score in the regression analysis. *p*-values < 0.05 were considered statistically significant.

Statistical analyses were conducted in the statistical program STATA (StataCorp. 2019. Stata Statistical Software: Release 16. College Station, TX, USA: StataCorp. LLC).

## 3. Results

The dataset had no missing values as the branching logic in the online research electronic data capture (REDCap) demanded complete registration for inclusion into the study.

A total of 607 persons accessed the survey link. Two-thirds of these (*n* = 417) completed all questionnaires (9 men, 211 women with AN, and 190 female controls). The male respondents were excluded from the current study, leaving 401 participants.

Participant characteristics and comparisons are reported in Table 1. Controls were slightly older than the women with AN and, as expected, had significantly higher BMI. Among the women with AN aged 13–18 years, 58% had a self-reported BMI-for-age percentile under 10%, indicating underweight.

Compared to the controls, the women with AN had significantly impaired disease-specific HRQoL as measured by the EDQLS total score (Table 1) and the EDQLS subscale scores (Table 2). Women with AN also had worse health status (SF-36), higher eating disorder psychopathology (EDI-3), more symptoms of depression (BDI), poorer wellbeing (WHO-5), and higher burden of functional impairment (WSAS) (Table 1).

Multiple regression analyses were conducted separately for women with AN and controls to identify factors associated with the EDQLS score. Both regression models explained 79% of the variance (R^2^ = 0.79). The models included the total scores for the EDI-3, BDI, WSAS, and WHO-5 as well as the scores for the eight subscales of the SF-36 and the clinical variables of age and BMI (the AN model also included nadir BMI, maximum BMI, and duration of disease).

Model I for the women with AN showed that EDI-3 total score, BDI total score, the SF-36 subscale scores for Role emotional and Vitality, and patient age were significantly associated with the total EDQLS score (Table 3). Model II for controls showed that only EDI-3 total score and the SF-36 subscale score for General health had significant association with the total EDQLS score.

We next examined which factors of each EDI-3 subscale were associated with the EDQLS total score. After controlling for depression (BDI) score, we found that for women with AN, four of the EDI-3 subscales were significantly associated (drive for thinness, bulimia, interpersonal insecurities, and maturity fears) (Table 4). For controls, only three of the EDI-3 subscales showed significant association with EDQLS score (drive for thinness, personal alienation, and interpersonal insecurities). These regression models explained 77% of the variance in EDQLS score for the patients with AN and 76% for controls.

## 4. Discussion

The study results show that women with AN reported significantly impaired function compared to healthy, normal-weight controls on all aspects measured, i.e., disease-specific HRQoL (EDQLS total score and all subscale scores), generic health status (SF-36), eating disorder psychopathology (EDI-3), depressive symptomatology (BDI), psychological wellbeing (WHO-5), and work functioning (WSAS). Further, we found that poorer disease-specific HRQoL in AN was associated with higher EDI-3 score, poorer psychological health (BDI score and SF-36 Role emotional and Vitality), and older age. In contrast, poorer HRQoL in controls was only associated with EDI-3 total score and the SF-36 subscale score for General health.

The current study confirms the significantly impaired HRQoL measured by SF-36 and EDQLS in women with AN, as reported previously [6,7]. Our finding that severe psychopathology in terms of eating disorders symptoms and depression was associated with poorer disease-specific HRQoL is in line with AN studies assessing generic HRQoL with SF-36 [31] and EQ VAS [32]. While several identical subscales of the EDI-3 were associated with poorer HRQoL in both women with AN and controls, impaired HRQoL in AN was also associated with bulimia and fear of reaching adulthood, as well as low levels of vitality (SF-36) and limitations in everyday life due to emotional problems (SF-36). These may be particularly important aspects of AN to consider during treatment, as they are likely to contribute to low motivation for completion of treatment. Jones et al. [33] suggested that motivation may be important for treatment completion, with completers being more motivated to improve their symptoms and their general quality of life.

Our regression model showed that impaired HRQoL was associated with higher age in women with AN. In the general population, SF-36 scores for physical health decreased with age, while SF-36 scores for mental health showed no clear age pattern [34]. Age has not previously been identified as an explanatory factor for disease-specific HRQoL in AN [14], although higher age has been associated with poorer clinical outcome in AN [35,36]. In population-based studies, aging itself has been perceived to decrease QoL, but this effect tends to diminish when controlled for other factors [37].

It is noteworthy that eating disorder pathology measured by EDI-3 was associated with HRQoL in both women with AN and controls. The EDI-3 measures psychological constructs that are clinically relevant in individuals with eating disorders, and the controls were not expected to exhibit eating disorder behavior. Eating disorders are likely to be present as a continuum, however, and include milder forms of disordered eating that would be present in a general population—and particularly in a young, female control group as in the current study. In future studies, it would be useful to investigate differences in HRQoL between people with a diagnosed ED, people with elevated ED symptoms but not a diagnosis, and people with non-disordered eating. The validity and reliability of applying the EDI-3 to healthy people has been questioned [38,39], as it has been designed specifically for individuals with eating disorders. When we used regression analysis to further investigate the explanatory value of the EDI-3 subscales, we found similar results for women with AN and controls (after controlling for depression) in terms of drive for thinness and interpersonal insecurities. However, bulimia and maturity fears were additionally associated with poorer HRQoL only in women with AN. Fear of adulthood/maturing is an inherent feature of people with AN and appears to play a significant role in determining self-reported HRQoL. While low individual body weight, characterized in drive for thinness and bulimia, may be a means of coping with the psychological conflict and imbalance of an eating disorder, the psychopathology in terms of emotional dysregulation and personal alienation may be consequences of the disorder. However, these factors may also very well be present in young females not diagnosed with an eating disorder but simply going through the tumultuous time of adolescence/early adulthood. When these different factors occur together, they appear to be related to a particularly low quality of life.

Comorbid mental disorders are common in AN and can require more intensive management of AN [40] as well as contribute to poorer outcome in terms of weight gain [41]. Our study participants were not asked about diagnosed comorbidities, but the medication use in the AN group indicated high psychiatric comorbidity. Among women with AN, 31% (*n* = 65) reported daily use of medication, with antidepressants representing almost half of this (*n* = 30). Ten of these were taking daily antipsychotic medication, while the remaining 25 women took daily medication for a somatic disorder (e.g., diabetes, thyroid disorder). Study participants with AN reported a significantly higher level of depressive symptoms compared to controls, with median scores indicating severe depression. A recently published scoping review found that comorbid depression was a negative predictor of prognosis in AN [40].

As expected, women with AN had a significantly lower median BMI compared to healthy controls, as well as a lower BMI-for-age percentile. However, about 42% of the 13–18 year olds with AN had a BMI-for-age percentile in the normal range despite being diagnosed with AN and in treatment at specialized centers for AN. Thus, the participants in our study comprised patients at very different stages of AN. We found no significant association between BMI (including BMI-for-age) and HRQoL in any of the regression models. This is in line with Abbate-Daga et al. [31], who found that eating disorder symptomatology but not BMI had an impact on HRQoL. It is in contrast, however, to the findings of Bamford et al. [13], who reported that changes in BMI predicted improvement in HRQoL. The latter study comprised a different and smaller selection of AN individuals than our study, as it included 63 adults with severe and enduring AN. This might explain the difference in results as patients with severe and enduring AN would be expected to have a higher burden of eating disorder symptomatology and to be more physically affected by their emaciated state, thus lowering their HRQoL. The abovementioned findings contribute to the argument that BMI cannot stand alone in determining outcome, but it is important to include PRO measures. It could be used in monitoring treatment response. In previous studies, nadir BMI has been determined to be a strong predictor of mortality/poor outcome in AN [42], but in this study, BMI was not associated with HRQoL, which needs to be considered when assessing outcome.

The strengths of our study are that it represents the largest AN cohort study to date, includes a wide range of assessment measures, and collects data from treatment-seeking individuals in all regions of Denmark. The analyses are further strengthened by the absence of missing values on the assessment measures. A limitation of the current study is that the controls were significantly older than the women with AN (median 24 years vs. 22 years). Recruitment of controls was achieved by advertising online through social media, and the results reflect self-selection. Thus, the control group is perhaps best described as normal-weight women without self-reported psychiatric disorders who did not take regular medication. This recruitment approach also meant that we did not know how many potential participants started the questionnaire but did not complete it. Furthermore, the study relies on self-reported data and lacks information about psychiatric comorbidity in the AN sample, and some may suffer from psychotic symptoms, since they were treated with antipsychotic medication. While some patient participants may have had a comorbid psychiatric diagnosis, they had all been diagnosed with AN and were in treatment for this.

Another limitation was that BMI was calculated from self-reported weight and height. In the literature, there is conflicting data about the validity of this. However, in a recent study of 100 healthy young adults, good agreement was detected between self-reported and direct anthropometric measurements [43], and to our knowledge, it has only been investigated exclusively in one study of AN. In a small population of AN patients (n = 23), self-reported weight and height were not significantly different from actual measured values, in contrast to differences found for controls and patients in remission from AN [44].

Some of the patients with AN were under treatment with different medicines, e.g., for anxiety, depression, or insomnia; in total, 65 individuals out of 187 (35%). Medication in itself may have influenced the results either in a negative or positive direction. The objective of medical treatment with psychotropics in AN is to reduce the effect of comorbid disorders, so it could be assumed that individuals treated with psychotropics were mostly benefitting from this treatment. Another factor that may have influenced the results is that the total BDI score was somewhat higher in AN patients with medication (mean BDI 35) compared to those without medication (mean BDI 30; *p* = 0.004). This supports the finding that depression influenced the association between eating disorder symptomatology and HRQoL.

The results of this study would be useful as reference material for intervention studies where EDQLS is included as an effect parameter. We further hope that the study results will help stimulate interest in patient-reported outcome measures in AN, which presents very special challenges in the form of patient ego-syntonicity and ambivalence. Biomedical effect measures cannot stand alone in AN, as “successful” treatment in terms of anthropometric and biomedical effect parameters can also be associated with reduced patient-reported quality of life.

## 5. Conclusions

Compared to similarly aged, normal-weight female controls, women in treatment for AN had a profoundly impaired quality of life, both in terms of disease-specific HRQoL and on measures of general health, psychological wellbeing, and work functioning. Poor disease-specific HRQoL in women with AN was associated with more severe eating disorder symptoms, poorer psychological health (depression, emotional role, and vitality), and older age. Bulimia and maturity fears were identified as factors associated with poorer HRQoL in women with AN but not in normal-weight controls. Most of these factors are psychological and cognitive in nature, emphasizing that management of disordered self-assessment and thought processes may be of particular importance to women with AN. It is possible that greater emphasis on these aspects alongside weight gain would enhance patient–clinician alliance and contribute to better treatment outcome. Future studies should focus on comparisons between diverse eating disorder diagnoses in large samples to better understand how disease-specific quality of life may differ in AN, bulimia nervosa, and binge-eating disorder.

## Figures and Tables

**Table 1:** Characteristics of women with anorexia nervosa (AN) and normal-weight controls. Data are medians (with interquartile range) or per cents.

	Women with AN (*n* = 211)	Controls (*n* = 190)	*p*-Value
Age (years)	21.7 (7.7)	23.9 (8.6)	0.001
BMI ^1^ (kg/m^2^)	16.5 (2.1)	21.7 (1.9)	0.000
Nutritional status; normal BMI, N (%) ^2^	4 (3.2%)	138 (100%)	0.000
13–18 s with low BMI ^3^, N (%)	50 (58%)	0	0.000
Nadir BMI (kg/m^2^)	14.5 (2.1)	n/a	n/a
Maximum BMI (kg/m^2^)	22.1 (6.7)	n/a	n/a
On medication (%)	31	0	0.000
Currently using drugs (%)	1.9	0.5	0.20
Current alcohol user (%)	0.5	0	0.33
Duration of AN (years)	5.5 (6.7)	n/a	n/a
EDQLS total score	99 (25.7)	160.7 (21.1)	0.000
SF-36 subscale scores: Physical functioning	78.5 (20.8)	95.9 (9.2)	0.000 ^4^ <0.05
Role physical	52.5 (32.8)	89.8 (16.5)	<0.05
Bodily pain	63.3 (24.8)	83.2 (19.7)	<0.05
General health	50.1 (22.2)	79.4 (18.2)	<0.05
Vitality	28.9 (21.8)	61.6 (19.4)	<0.05
Social functioning	42.2 (27.1)	87.6 (18.0)	<0.05
Role emotional	46.2 (30.4)	81.6 (21.1)	<0.05
Mental health	37.1 (19.4)	73.7 (16.1)	<0.05
EDI-3 total score	174.2 (57.5)	70.6 (51.6)	0.04
BDI total score	31.4 (12.9)	7.2 (8.1)	0.000
WHO-5 total score	38.4 (18.8)	69.4 (16.1)	0.009
WSAS score	23.2 (9.6)	4 (6.8)	0.000

^1^ BMI values are calculated from self-reported weight and height. ^2^ For adults only (19 years and over). ^3^ 13–18 year olds with BMI-for-age under 10th percentile. ^4^ For all SF-36 subscales combined. Abbreviations: BMI, body mass index; EDQLS, Eating Disorders Quality of Life Scale; SF-36, Short-Form 36; EDI-3, Eating Disorder Inventory-3; BDI, Beck Depression Inventory; WHO-5, World Health Organization wellbeing index; WSAS, Work and Social Adjustment Scale; n/a: not applicable.

**Table 2:** Median EDQLS subscale scores for women with anorexia nervosa (AN) and normal-weight controls. Data are medians (with interquartile ranges).

	Women with AN (*n* = 211) ^1^	Controls (*n* = 190)
EDQLS subscales		
Cognitive	7.7 (2.6)	12.6 (1.8)
Education	6.9 (2.8)	13.3 (1.8)
Family/close relationships	8.6 (2.6)	13.2 (1.9)
Relationships with others	7.8 (2.6)	12.3 (2.3)
Future/outlook	9.7 (3.1)	13.6 (1.7)
Appearance	6.4 (2.3)	9.9 (2.4)
Leisure	9.3 (2.3)	13.0 (1.6)
Psychological	7.8 (2.8)	12.0 (2.1)
Emotional	6.3 (2.3)	10.8 (2.4)
Values and beliefs	6.4 (2.5)	10.4 (2.5)
Physical	6.8 (2.8)	11.3 (2.3)
Eating disorders	15.2 (5.8)	28.3 (4.9)

^1^ All AN values were significantly lower than control values (all *p* = 0.000). Abbreviations: EDQLS, Eating Disorders Quality of Life Scale.

**Table 3:** Multiple regression analyses with bootstrapped standard errors (500 replications) showing effect of independent variables on EDQLS total score for women with anorexia nervosa (AN).

	Model I (*n* = 211) Women with AN		Model II (*n* = 190) Controls	
	Coefficient ^1^	*p*-Value	Coefficient ^1^	*p*-Value
EDI-3 total score	−0.21	<0.001	−0.21	<0.001
BDI total score	−0.42	0.004	−0.22	0.37
WHO-5 total score	0.07	0.5	−0.02	0.37
WSAS score	−0.28	0.11	0.14	0.52
SF-36 subscale scores: Physical functioning	0.08	0.11	−0.04	0.74
Role physical	−0.01	0.86	0.13	0.14
Bodily pain	−0.06	0.3	−0.05	0.39
General health	0.08	0.16	0.15	0.03
Vitality	0.21	0.01	0.08	0.23
Social functioning	0.03	0.56	0.15	0.10
Role emotional	−0.14	<0.001	0.01	0.86
Mental health	0.01	0.9	0.17	0.06
Age	0.42	0.03	−0.14	0.1
BMI ^2^	−0.1	0.8	0.52	0.22
Nadir BMI	−0.33	0.61	-	-
Maximum BMI	0.11	0.57	-	-
Duration of AN	−0.34	0.06	-	-
R-squared	0.79		0.79	

^1^ The coefficients can be interpreted as follows: for every 1 unit increase in EDI-3 total score, for example, we can expect to see a negative 0.21 drop in EDQLS score. ^2^ BMI values are calculated from self-reported weight and height. Abbreviations: EDQLS, Eating Disorders Quality of Life Scale; EDI-3, Eating Disorder Inventory-3; BDI, Beck Depression Inventory; WHO-5, World Health Organization wellbeing index; WSAS, Work and Social Adjustment Scale; SF-36, Short-Form 36; BMI, body mass index.

**Table 4:** Multiple regression showing effect of EDI-3 scores on EDQLS total score, after controlling for depression (BDI score), for women with anorexia nervosa (AN) and normal-weight controls.

	Women with AN *n* = 211		Controls *n* = 190	
	Coefficient	*p*-Value	Coefficient	*p*-Value
EDI-3 subscales: Drive for thinness	−0.91	0.000	−0.76	0.001
Bulimia	0.32	0.02	0.04	0.86
Body dissatisfaction	−0.13	0.37	−0.13	0.32
Low self-esteem	−0.39	0.18	0.02	0.96
Personal alienation	−0.40	0.14	−0.88	0.02
Interpersonal insecurities	−0.52	0.003	−0.66	0.01
Interpersonal alienation	0.07	0.76	0.42	0.22
Interoceptive deficits	−0.04	0.78	0.03	0.88
Emotional dysregulation	−0.26	0.19	−0.33	0.21
Perfectionism	−0.15	0.39	−0.25	0.18
Ascetism	−0.06	0.79	−0.33	0.33
Maturity fears	−0.81	0.03	0.09	0.52
R-squared	0.77		0.76	

Abbreviations: EDI-3, Eating Disorder Inventory-3; EDQLS, Eating Disorders Quality of Life Scale.

## Data Availability

The data presented in this study are available on request from the corresponding author. The data are not publicly available due to legal restrictions.
